# Modeling of COVID-19 Outbreak Indicators in China Between January and June

**DOI:** 10.1017/dmp.2020.323

**Published:** 2020-09-09

**Authors:** Senol Celik, Handan Ankarali, Ozge Pasin

**Affiliations:** Department of Biometry and Genetics, Faculty of Agriculture, Bingol University, Bingol, Turkey; Department of Biostatistics and Medical Informatics, Faculty of Medicine, Istanbul Medeniyet University, Istanbul, Turkey; Department of Biostatistics, Faculty of Medicine, Istanbul University, Istanbul, Turkey

**Keywords:** ARIMA, coronavirus, exponential smoothing, nonlinear model

## Abstract

**Objectives:**

The objective of this study is to compare the various nonlinear and time series models in describing the course of the coronavirus disease 2019 (COVID-19) outbreak in China. To this aim, we focus on 2 indicators: the number of total cases diagnosed with the disease, and the death toll.

**Methods:**

The data used for this study are based on the reports of China between January 22 and June 18, 2020. We used nonlinear growth curves and some time series models for prediction of the number of total cases and total deaths. The determination coefficient (R^2^), mean square error (MSE), and Bayesian Information Criterion (BIC) were used to select the best model.

**Results:**

Our results show that while the Sloboda and ARIMA (0,2,1) models are the most convenient models that elucidate the cumulative number of cases; the Lundqvist-Korf model and Holt linear trend exponential smoothing model are the most suitable models for analyzing the cumulative number of deaths. Our time series models forecast that on 19 July, the number of total cases and total deaths will be 85,589 and 4639, respectively.

**Conclusion:**

The results of this study will be of great importance when it comes to modeling outbreak indicators for other countries. This information will enable governments to implement suitable measures for subsequent similar situations.

The novel coronavirus disease 2019 (2019-nCoV, or COVID-19) epidemic first broke out in Wuhan, China.^1,2^ The virus was identified in the second half of December 2019.^3^ The epidemiological features of the disease are still unknown, and the number of total cases and deaths varies daily. In the wake of its rapid spread and reports revealing the crucial consequences of this spread, countries adopted strict measures to tackle the disease. However, confirmed positive cases were recorded after the second half of January 2020. Mathematical models used to identify the quantitative description of the outbreak of COVID-19 in this study may provide significant insight into the cessation of the spread of the novel coronavirus.^4^ Various indicators are used in the models to describe the course of the outbreak. Among these indicators, the total number of confirmed cases and the total number of deaths are the most commonly used ones.

The objective of this study is to compare the various nonlinear and time series models in describing the course of the COVID-19 outbreak in China. To present this, we focused on 2 indicators: the number of total cases diagnosed with the disease and the number of deaths.

## METHODS

### Data Management

We obtained daily updates of the cumulative number of reported confirmed cases and deaths for the 2019-nCoV pandemic of China between January 22 and June 18, 2020, from Worldometer and WHO websites.^5,6^ In this study, we focus on China. Because it is not only the country where the novel coronavirus emerged and has spread throughout the world but is also the country that has been fighting against the coronavirus for the longest time. Also, due to unpreparedness for the outbreak, the studied period can be observed as a sample of natural course, especially in the first month.

### Models for Describing the Course of the Outbreak

The models that we apply for the abovementioned indicators can be categorized into 2 categories: (1) nonlinear growth curves including the Weibull, negative exponential, Von Bertalanffy, Janoscheck, Lundqvist-Korf, and Sloboda models (Table 1)^7-13^; and (2) time series models including Box-Jenkins and exponential smoothing methods (Tables 2 and 3).

**TABLE 1:** Nonlinear Growth Curves

Model	Equation
Weibull model	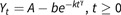
Negative exponential model	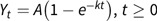
Von Bertalanffy model	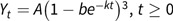
Janoscheck model	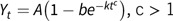 c > 1
Lundqvist-Korf model	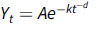
Sloboda model	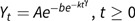

**TABLE 2:** Box-Jenkins Models

Model	Equation
Autoregressive model (AR(p))	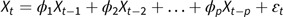
Moving averages model (MA(q))	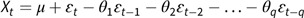
Autoregressive moving averages model (ARMA(p,q))	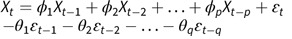

**TABLE 3:** Exponential Smoothing Models

Model	Equation
Holt double exponential smoothing model	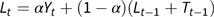 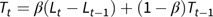 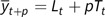
Damped trend model	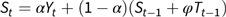 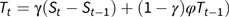 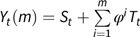
Brown’s single parameter linear exponential smoothing model	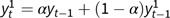 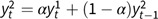 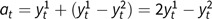 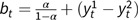 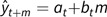

*Y*_*t*_ is the observed dependent variable named the number of total cases and the number of total deaths, and *t* is the independent variable named as time (Table 1). In our models, t is the day. The *A* term is the asymptotic limit of the number of total cases and the number of total deaths as time goes to infinity; *B* is the proportion of the number of total cases to the number of total deaths. The *k* term is the proportion of the maximum increase rate to the highest number of cases or deaths. *γ*, *c*, and *d* are the changing points that occur when the change in the estimated increase rate goes from increase to decrease.

AR (*p*) is the *p* ^th^ degree of autoregressive series.^14^ MA (*q*) refers to the moving average model of order *q*. In this series, *ε_t~WN(0,σ* ^*2*^ *)* is the white noise series.^15^ The ARMA(*p, q*) model is expressed by both AR (*p*) and MA (*q*) processes.^16^

In the Holt method, *L*_*t*_ is the new smoothed value, *α* is the smoothing coefficient, (0 < *α* < 1), *Y*_*t*_ is the actual value at *t* ^th^ period, *β* is the smoothing coefficient for trend estimation, (0 < *β* < 1), *T*_*t*_ is the trend predicted value, *p* is the number of forecasting periods and 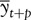
is the forecasting value after *p* period.^17^ In the damped trend method, if 0 < *φ* < 1, the trend is damped, if *φ* = 1, the equations become identical to Holt’s linear trend method. Tashman and Kruk (1996) suggested that there may be value in allocating *φ* > 1, if applied in a series with a strong tendency, with exponential trend.^18^ The Brown’s single parameter linear exponential smoothing model is more suitable if there is an increasing or decreasing trend in the time series. In this model, the initial equations 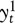 and 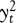 are obtained by single exponential smoothing and double exponential smoothing, respectively.^19^ For the estimation of post m process, the equation is given below.^20^

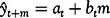



In exponential smoothing methods, the estimations are constantly updated, taking into account recent changes in data.^21^ In these methods, the weighted average of past period values is calculated and taken as the estimated value of future periods.

Estimation accuracy of the applied methods were evaluated with BIC, R^2^ and MSE. BIC was developed by Gideon E. Schwarz (1978), who gave a Bayesian argument for adopting it.^22^

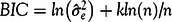



Where 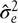 is the error variance

## RESULTS

### Results of Nonlinear Growth Models for the Number of Total Cases

The parameters estimated and goodness of fit measures of the nonlinear models between January 22 and June 18, 2020, in China were presented in Table 4. R^2^ and MSE statistics were used to compare models. The R^2^ and MSE values of the Weibull and Janoscheck models were equal. The MSE of the Sloboda model was slightly smaller than the Weibull and Janoscheck models, but R^2^ was equal. The Sloboda model can be considered the most suitable model, as it has a smaller MSE value and a larger pseudo R^2^ value. The Weibull and Janoscheck models could also be chosen as alternative models.

**TABLE 4:** Parameters Estimated and Goodness of Fit Measures of the Nonlinear Models for the Number of Total Cases

Model	A	b	k		MSE	**R** ^2^
Weibull	82971.8	81813.8	0.00028	γ = 2.672	2923769.5	0.995
Negatif exponential	88954.9		0.042		72795574.9	0.903
Von Bertalanffy	83770.1	1.414	0.109		5346914.3	0.991
Janoschek	82971.8	0.986	0.00028	c = 2.672	2923769.5	0.995
Lundqvist-Korf	84441.2		2826.493	d = −2.846	6084525.2	0.990
Sloboda	83091.3	4.150	0.022	γ = 1.51	2689232.2	0.995

**FIGURE 1:**
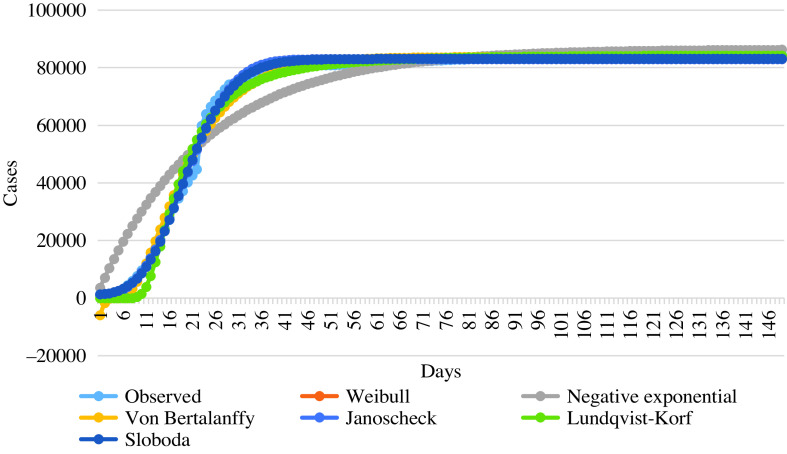
Curve for Prediction of Nonlinear Models for the Number of Total Cases.

The curve for prediction of nonlinear growth models are given in Figure 1.

### Results of Time Series Models for the Number of Total Cases

**FIGURE 2:**
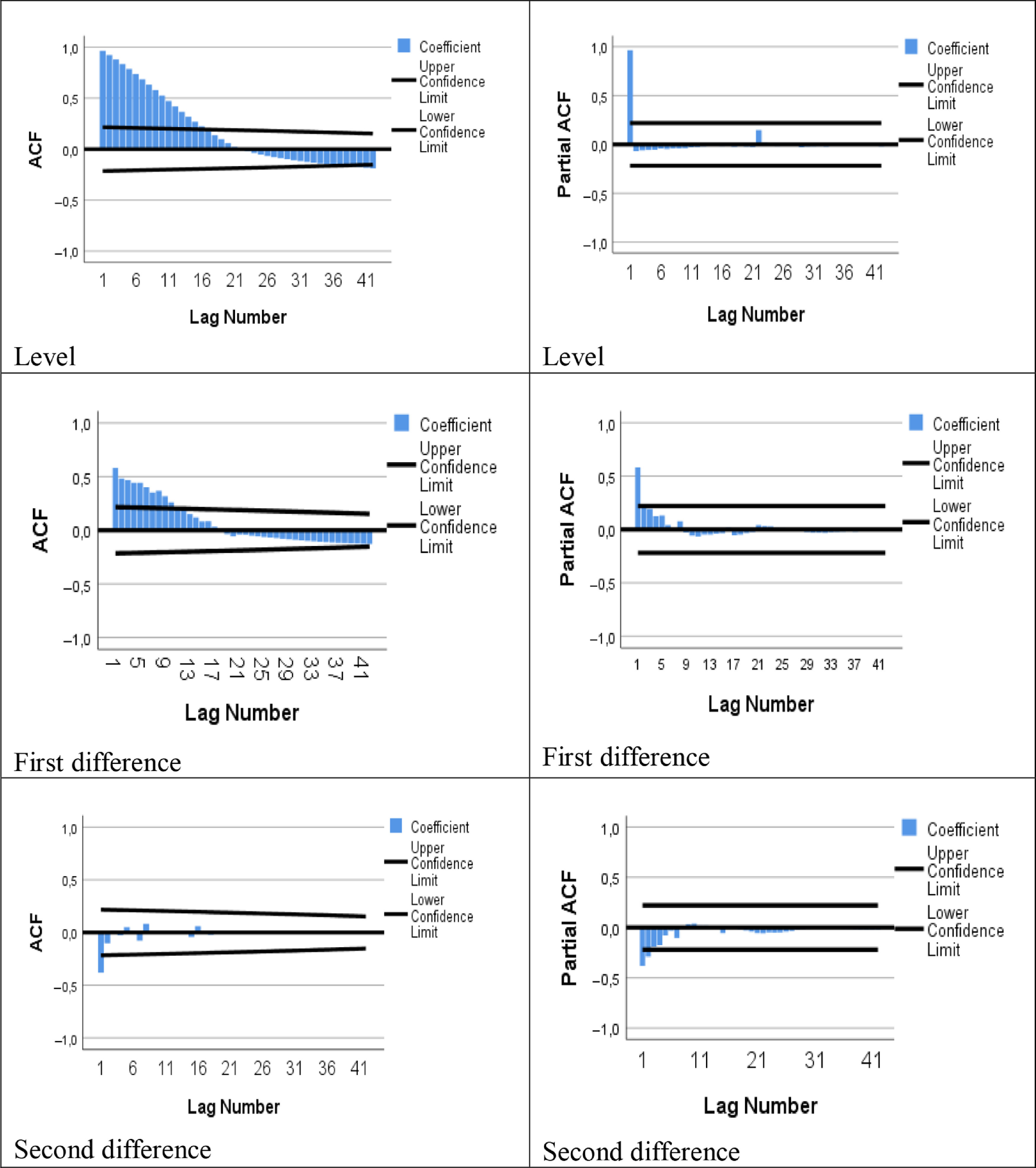
ACF and PACF Graphs for the Number of Total Cases.

Box-Jenkins and exponential smoothing methods were chosen from the various time series models available for the total number of cases. Autocorrelation (ACF) and partial autocorrelation (PACF) graphs of the series were examined. When the ACF and PACF graphs in Figure 2 were examined, the first degree difference was taken because the series was not stationary at that level. But the stationary assumption had not been provided yet. The difference from the second degree was taken and the series became stationary. According to the ACF and PACF charts, the series quickly approached zero after the first delay in the ACF graph. In this case, because *p* = 0, *d* = 2, and *q* = 1, it was modeled by the integrated first degree moving averages method. In other words, the most suitable time series method was the ARIMA(0,2,1) model. In addition, exponential smoothing methods were used and the model performances were given in Table 5.

**TABLE 5:** Goodness of Fit Measures of the Time Series Models for the Number of Total Cases

Model	**R** ^2^	BIC	Ljung-Box
ARIMA(0,2,1)	0.997	14.183	3.685 and p = 0.999
Holt	0.998	14.209	3.714 and p = 0.999
Brown	0.997	14.206	9.119 and p = 0.936
Damped	0.998	14.229	3.633 and p = 0.999

The performance of the ARIMA(0,2,1) model is given in Table 6, and it is observed that this model’s fits are successful as nonlinear models.

**TABLE 6:** Goodness of Fit Measures of the ARIMA(0,2,1)

Model Fit statistics				Ljung-Box Q		
**Stationary R-squared**	**R-squared**	**RMSE**	**Normalized BIC**	**Statistics**	**DF**	**p**
0.321	0.997	1181.472	14.183	3.685	17	0.999

The parameters estimated of the ARIMA(0,2,1) model are given in Table 7.

**TABLE 7:** Parameters Estimated of the ARIMA(0,2,1)

	Estimate	SE	t	***P*** **-Value**
Difference	2			
MA(1)	0.707	0.059	12.002	0.001

The ARIMA(0,2,1) model was found to be the most appropriate among different time series models. This model can be written as follows:
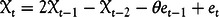


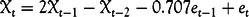



Forecasting data for future 30 d are given in Table 8.

**TABLE 8:** Forecasting Data for Future 30 Days According to ARIMA(0,2,1)

Date (June 19- July 18)	Days	Case Forecasting	Case Actual
June 19	151	84530	84524
June 20	152	84567	84553
June 21	153	84603	84572
June 22	154	84640	84624
June 23	155	84676	84653
June 24	156	84713	84673
June 25	157	84749	84701
June 26	158	84786	84725
June 27	159	84822	84743
June 28	160	84859	84757
June 29	161	84895	84780
June 30	162	84932	
July 1	163	84968	
July 2	164	85005	
July 3	165	85041	
July 4	166	85078	
July 5	167	85114	
July 6	168	85151	
July 7	169	85187	
July 8	170	85224	
July 9	171	85260	
July 10	172	85297	
July 11	173	85333	
July 12	174	85370	
July 13	175	85406	
July 14	176	85443	
July 15	177	85479	
July 16	178	85516	
July 17	179	85552	
July 18	180	85589	

**FIGURE 3:**
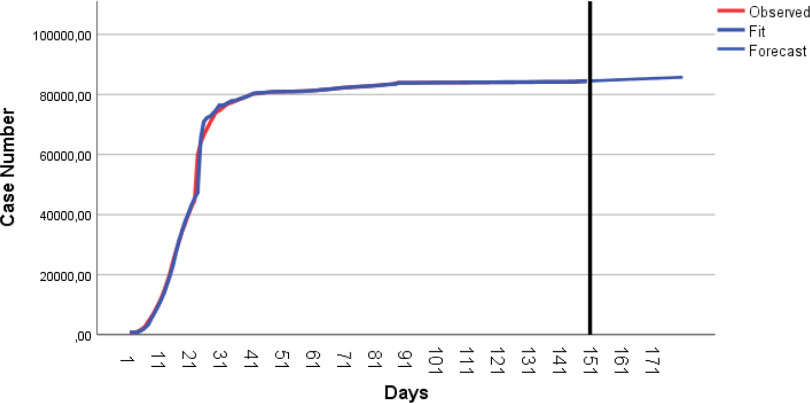
Observed and Predicted Values for the Number of Total Cases by ARIMA(0,2,1).

The number of total cases continues increasingly, albeit at a low speed. The number of total cases is predicted to be 85,589 on July 18, 2020 (Table 8). Observed and predicted values of the total cases are given in Figure 3.

### Results of Nonlinear Growth Models for the Number of Total Deaths

**FIGURE 4:**
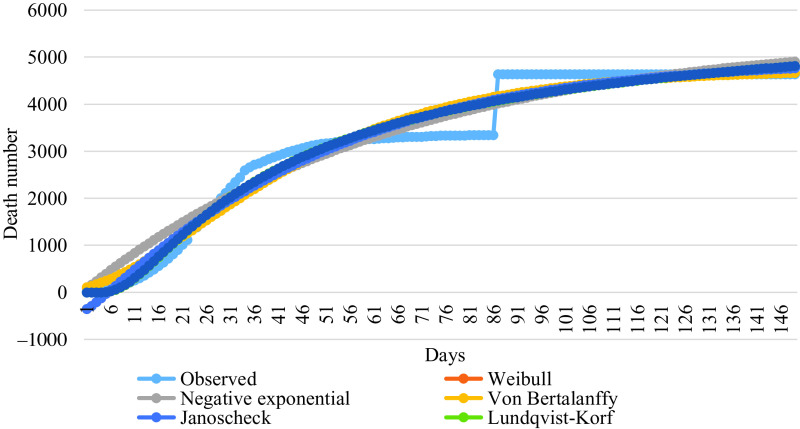
Curve for Prediction of Nonlinear Models for the Number of Total Deaths.

The parameters estimated and goodness of fit measures of the nonlinear models for the number of total deaths are presented in Table 9. The most suitable models for predicting the number of total deaths are the Lundqvist-Korf and Sloboda models, respectively (Table 9). The R^2^ values of these models were found to be the highest at 0.963 and also the MSE values of these were lower than the others. The Lundqvist-Korf model can be considered the most suitable one, because mean square error (MSE) is smaller than other models. The curve for prediction of nonlinear models are given in Figure 4.

**TABLE 9:** Parameters Estimated and Goodness of Fit Measures of the Nonlinear Models for the Number of Total Deaths

Model	A	b	k		MSE	**R** ^2^
Weibull	4957.440	5313.784	0.014	γ = 1.099	93529.037	0.959
Negative exponential	5486.157		0.015		116911.341	0.948
Von Bertalanffy	4747.165	0.715	0.033		105015.273	0.953
Janoschek	4957.440	1.072	0.014	c = 1.099	93529.037	0.959
Lundqvist-Korf	6125.155		30.841	d = −0.969	83257.444	0.963
Sloboda	5929.037	158799.877	9.047	γ = 0.081	84125.689	0.963

### Results of Time Series Models for the Number of Total Deaths

The most suitable time series model was found to be the Brown linear trend exponential smoothing model among time series models for the number of deaths. The goodness of fit of the various models are given in Table 10, and it was observed that the predictions are as successful as nonlinear models.

**TABLE 10:** Goodness of Fit Measures of the Time Series Models

Model	**R** ^2^	BIC	Ljung-Box
ARIMA(1,1,0)	0.994	9.497	4.389 and p=0.999
ARIMA(0,1,1)	0.994	9.498	4.626 and p=0.999
ARIMA(1,1,1)	0.994	9.520	0.239 and p=0.999
Holt	0.994	9.495	6.229 and p=0.985
Brown	0.994	9.454	10.541 and p=0.879
Damped	0.994	9.533	5.696 and p=0.984

**FIGURE 5:**
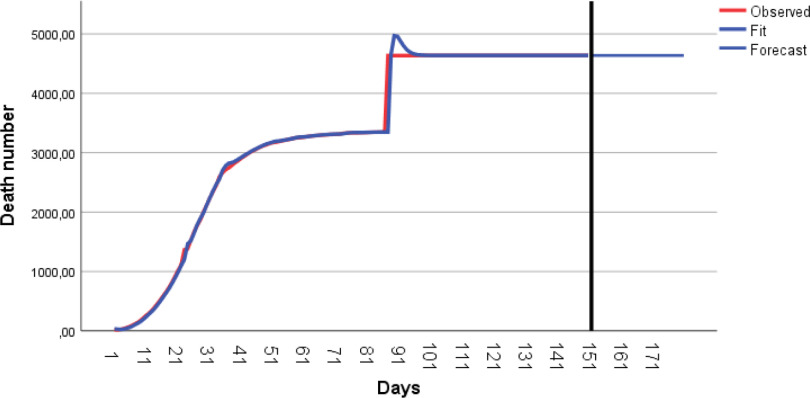
Curve for Prediction of the Brown Linear Trend Exponential Smoothing Model for the Number of Total Deaths.

The parameters estimated of the Brown linear trend exponential smoothing model are presented in Table 11. The observed and predicted values are given in Figure 5.

**TABLE 11:** Parameters Estimated of the Brown Linear Trend Exponential Smoothing Model

	Estimate	SE	t	***P*** **-Value**
Alpha (Level)	0.509	0.036	14.268	0.001

The forecasts of the number of total deaths using Holt’s linear trend exponential smoothing model for 30 d are given in Table 12. The rate of increase in the number of deaths in China was decreasing, and it was predicted that the number will be between 3343 and 3355 in the period between June 19 and July 18, with a slight increase (Table 12). The Holt linear trend exponential smoothing curve for the exponential smoothing model is given in Figure 5.

**TABLE 12:** Forecasting Results for Brown Exponential Smoothing Model

Date (June 19- July 18)	Days	Deaths Forecasting	Death actual
June 19	151	4639	4638
June 20	152	4639	4639
June 21	153	4639	4639
June 22	154	4639	4639
June 23	155	4639	4640
June 24	156	4639	4640
June 25	157	4639	4641
June 26	158	4639	4641
June 27	159	4639	4641
June 28	160	4639	4641
June 29	161	4639	4641
June 30	162	4639	4641
July 1	163	4639	
July 2	164	4639	
July 3	165	4639	
July 4	166	4639	
July 5	167	4639	
July 6	168	4639	
July 7	169	4639	
July 8	170	4639	
July 9	171	4639	
July 10	172	4639	
July 11	173	4639	
July 12	174	4639	
July 13	175	4639	
July 14	176	4639	
July 15	177	4639	
July 16	178	4639	
July 17	179	4639	
July 18	180	4639	

## DISCUSSION

In this study, we found that the Sloboda model for the number of total cases and the Lundqvist-Korf model for the number of total deaths were the best explanatory models among the nonlinear models used in the study. Also, the ARIMA(0,2,1) model for the number of cases and the Brown linear trend exponential smoothing model for the number of deaths were the most suitable models among the time series models used in the study.

In a different study, the ARIMA model was used on the daily prevalence data of COVID-2019 from January 20, 2020, to February 10, 2020, and the ARIMA(1,2,0) and ARIMA(1,0,4) models were obtained.^23^ The logistics, Bertalanffy, and Gompertz models were previously used to estimate the number of cases and deaths from COVID-19 in different regions in China by Jia et al. (2020).^24^ In their study, the Logistics model was reported to be better than the others. They conducted an extensive research with quasi-experimental analysis methods in various provinces in China and investigated the relationship between population and the number of outbreak cases. Accordingly, they found that the correlation coefficients of the relationship between the population and the number of cases differed by regions. They observed that the number of cases was higher in regions with high populations and that there was a high correlation between them. They concluded factors such as immigration, tourism, and mobility play an important role in this situation. The authors also determined the number of cases using the epidemic growth model.^24,25^

On the other hand, Roosa et al. (2020) analyzed the number of cases in some regions of China using the generalized logistic growth model (GLM), the Richards Model and the sub-epidemic model for a short time period (10 d). They found that the number of confirmed cases will continue to increase. They estimated that the predicted case increase (GLM) in the Guangdong and Zhejiang regions would be lower by using the Richards models and that it would be higher using the sub-epidemic model.^26^

In a study on the risk of infection when COVID-19 was detected in a cruise ship in China in February 2020, it was noted that the risk of infection among people who have close contact was higher than those who maintained a social distance from others. The estimated number of cases was obtained by the back-calculation method.^27^ Al-qaness et al. (2020) used the Adaptive Neuro-Fuzzy Inference System (ANFIS), the Flower Pollination Algorithm (FPA), the Salp Swarm Algorithm (SSA), and the FPASSA-ANFIS method to estimate the number of cases of COVID-19 in China and the United States. They calculated model performance using root mean square error (RMSE), mean absolute error (MAE), mean absolute percentage error (MAPE), root mean squared relative error (RMSRE), and R2. They found that the best method for modeling and estimating the number of total cases was the FPASSA-ANFIS method.^28^

Kuniya (2020) estimated the outbreak peak of coronavirus disease in Japan using the susceptible-exposed-infected-removed (SEIR) compartmental model.^29^ In another study, the reproduction number of the Wuhan novel coronavirus 2019-nCoV was estimated using the SEIR compartment model.^30^ There are many studies on coronavirus disease by various researchers using different statistical methods. Among these studies, the following are highlighted: Yuan et al. (2020) used the median (interquartile range, IQR) and Mann Whitney U test or Wilcoxon test. Twu et al. (2020), Prem et al. (2020), and Neher et al. (2020) used the SEIR model.^31-34^

In our study, we compared the time series analysis using the Weibull, negative exponential, Von Bertalanffy, Janoscheck, Lundqvist-Korf, and Sloboda models, which are different from the methods used in previous studies. Based on our extensive literature review, this study has been the first and most comprehensive study based on the nonlinear models as we discussed in detail.

## CONCLUSIONS

While some models are simple and give general results, some are complex and provide detailed information, but their results cannot be generalized.^4^ Models that were used in the initial phase of the outbreak can be misleading because of a lack of sufficient data. Therefore, short-term predictions should be made for the early stages of the epidemic, and the effects of any measures taken in this process must be taken into consideration by virtue of their results. As for the further stages of pandemics, different models can be used to understand biological systems and to develop models which can be used for the simulation for future similar situations. However, although model assumptions are mostly incompatible with real-world problems, they can capture general behavior and predict the rate of the spread of the outbreak. If large-scale behaviors of a system are correctly identified, certain details can be understood in terms of their impact on these behaviors. Statistical or data-based models that fit curves of the past temporal prevalence of a disease, do not make any assumptions about the internal mechanisms that a mathematical model provides and, hence, have become more popular in infectious diseases. Because the major use of these models is to fit past data and estimate the future, it can also be used for different patterns of the epidemic.

As a result of the literature review, it was observed that the Sloboda model and Lundqvist-Korf model, which gave the best results among the nonlinear models used in this study, have never been used for modeling COVID-19 outbreak indicators before. Our most recent forecasts remained relatively stable. This reflects the impact of the measures implemented by the China government, which likely helped to stabilize the pandemic. The forecasts presented here are based on the assumption that current mitigation efforts will continue. In addition, comparing with other modeling studies on COVID-19, results were obtained for longer periods. Therefore, the results in this study are more favorable in terms of comprehending the biological structure of the outbreak and producing preliminary information for possible similar conditions in the future.
